# Noise-induced hearing loss in the pre-industrial era: early contributions in *De Morbis Artificum* by Bernardino Ramazzini (1633–1714)

**DOI:** 10.1017/S0022215123001457

**Published:** 2024-01

**Authors:** M E Paladino, M Belingheri, R Mazzagatti, M A Riva

**Affiliations:** 1 School of Medicine and Surgery, University of Milano-Bicocca, Monza, Italy; 2 Occupational Health Unit, Fondazione IRCCS San Gerardo dei Tintori, Monza, Italy

**Keywords:** History, otorhinolaryngology, otorhinolaryngologic diseases

## Abstract

**Objective:**

Noise-induced hearing loss is the most prevalent occupational disease worldwide and is historically associated with the Industrial Revolution. This study analyses early descriptions of this disorder during the pre-industrial period in the work of the Italian physician Bernardino Ramazzini (1633–1714).

**Method:**

Primary and secondary historical literature were reviewed.

**Results:**

Ramazzini described hearing loss in corn millers and in coppersmiths and recognised that this disorder is irreversible and progressive when exposure to noise continues. He also seemed to describe tinnitus. He further suggested the use of earplugs as a preventive measure for these classes of workers. Ramazzini's anatomical and pathological knowledge appears to be based on ancient authors; he did not discuss contemporaneous medical authors’ work on hearing function.

**Conclusion:**

Despite some limitations, Ramazzini's work appears pioneering for his time and represents an important milestone in the history of otolaryngology.

## Introduction

It is well established that continual exposure to loud sounds at the workplace can cause hearing disorders. Noise-induced hearing loss is the second most common cause of sensorineural hearing loss after age-related hearing loss and affects approximately 5 per cent of the population.^1^ This disorder is the most prevalent occupational disease worldwide. An estimated 16 per cent of adult hearing loss cases are associated with exposure to noise in the workplace, and more than 10 per cent of workers in developed countries may suffer from noise-induced hearing loss.^2,3^ Otolaryngologists can diagnose these disorders and work with occupational health physicians to define and facilitate workplace accommodations.

Noise-induced hearing loss is historically associated with the introduction of manufacturing machines powered from steam engines, starting with the Industrial Revolution.^4^ However, noise-induced hearing loss has been described in pre-industrial literature, particularly in the work of the Italian physician Bernardino Ramazzini, who is universally credited as the founder of occupational medicine.^5–7^

Ramazzini was born in Carpi, Italy, in 1633. After his medical graduation in 1659 in Parma, he worked as a physician in Rome and in the Duchy of Castro, a vassal state to the Papal States. In 1682, he was appointed Professor of Theoretical Medicine at the University of Modena by the Duke Francesco II d'Este (1660–1694).^8^ While in this role, he wrote several medical works, with a particular focus on population studies. At the end of the century, Ramazzini started to investigate the influence of occupations on workers’ health and taught a class on this subject at the University of Modena. He published the treatise *De Morbis Artificum* in 1700, which is the first publication on work-related health problems in different occupations.^9^ Each chapter of the treatise contains a description of the disease associated with a particular work activity followed by a literature analysis, workplace description, questions for the workers, disease description, remedies and advice.^10^ He analysed the effects of chemicals, dusts, repetitive motions and awkward postures on the health of different classes of workers and modernly sustained that rulers should protect the health of workers to preserve the workforce and the productivity of their states.^10^ In 1700, Ramazzini's renown enabled him to move to the University of Padua, where he was appointed as Professor of Practical Medicine.^9^ Towards the end of his life, he continued to teach students and published a new edition of *De Morbis Artificum* (1713), which included new occupations. He died in Padua in 1714, but his work had an immediate and lasting impact, inspiring the studies on occupational health in the following centuries.^8^

### Noise-induced hearing loss in coppersmiths and corn millers

In Chapter XXII (‘Diseases of bakers and millers’) of *De Morbis Artificum*, Ramazzini reported that corn millers often suffered from hearing loss. In particular, he wrote, ‘nearly all of them are half-deaf [*surdastri*] because they spend all night and day surrounded by the repetitive noise of wheels and millstones and the roar of water falling from a height; the tympanum, subjected to more powerful noise than it can tolerate and unremitting blows loses its tone’.^8^ Another accurate description of the effects of exposure to noise in the workplace can be found in the second edition of *De Morbis Artificum*, dated back to 1713. Regarding the diseases of coppersmiths (Chapter V), Ramazzini stated:

one can observe these workers […] bent over all day hammering, first with wood, then with iron, working the newly mined copper until it is as ductile as required. It is obvious that this constant din hurts both their ears and heads. In fact, these workers become half deaf [*surdastri*] and, if they continue this profession, stone-deaf [*et, ubi in hoc opera consenuerint, omnino surdi*]. Because of that constant percussion, the tympanum of the ear loses its natural tension and the repercussion of the internal air [*aeris interni*] towards the outside weakens and subverts the entire hearing apparatus [*auditus organa*].^8^

These texts demonstrated that Ramazzini was aware of the effects of noise on hearing, distinguishing hearing loss (*surdastri*) from deafness (*surdi*). He also recognised that this disorder is irreversible and progressive while the exposure to noise continues (‘if they continue this profession’). Ramazzini believed that hearing loss occurred when the tympanum was damaged by constant loud sound, reducing its natural tension and rendering it ‘weak’. The Italian physician was apparently ignorant of studies on the ossicles of the middle ear by Renaissance anatomists (Vesalius, Falloppius, Eustachi and Ingrassia) and the works of his contemporaries, such as Antonio Maria Valsalva (1666–1723), who published his masterpiece *De aure humana tractatus* in 1704.^11,12^ This apparent lack of knowledge is not surprising; Ramazzini was an adherent of the neo-Hippocratism doctrine and followed the teachings of Hippocrates and other ancient physicians.^13^ This approach was inspired by the English physician Thomas Sydenham (1624–1689) and the French scholar Guillaume de Baillou (1538–1616). Ramazzini based his thinking on the doctrines of Hippocrates, who first described the tympanic membrane in his treatise *De carnibus*, and on those of Aristotle, who argued that audition took place within a completely closed ear cavity filled with air (*aer innatus*). The Aristotelian concept of *aer innatus* seems to be referenced by Ramazzini's use of the words *aer internus*.

In his accounts of the health conditions of coppersmiths, Ramazzini seems to describe tinnitus, a symptom that is strongly associated with hearing loss. As he stated, ‘the workers frequently complain of a ringing in their ears [*aurium sonitus*], which they believe to be a bad omen, but only because according to Hippocrates, such noises are a dangerous symptom. It is not at all surprising that the hearing of coppersmiths has been impaired and […] such noises are accentuated’.^8^

Finally, Ramazzini proved to be pioneering in his suggestion of preventive measures and treatment for hearing disorders in coppersmiths. He suggests that ‘their ear could be plugged with cotton to protect the internal parts and, when they are bruised and battered by the constant noise, they could be rubbed with sweet almond oil’.^8^

## Conclusions

Bernardino Ramazzini can be credited as the first author to accurately describe noise-induced hearing loss among specific classes of workers in the pre-industrial era. Although his knowledge remained anchored in ancient medicine and was not current with the discoveries in physiology and anatomy that occurred during the Renaissance, Ramazzini demonstrated unprecedented attention to workers’ suffering and suggested remedies and preventive measures such as archaic earplugs. Recently, Thurston criticised Ramazzini for not discussing the risk of blacksmiths becoming deaf ‘even though they were actively producing loud impact sounds with their hammer blows, and were surrounded by other noises common to smithies’.^4^ This criticism may be correct if one considers that Ramazzini was always very careful and detailed in his description of the diseases that affected workers. As noted in the present paper, Ramazzini added a chapter on the diseases of coppersmiths in the supplement of the second edition of *De Morbis Artificum*, published in Padua in 1713. Perhaps the Italian physician, aware of the serious oversight of hearing disorders in blacksmiths, added this chapter, dedicated to a numerically very limited number of workers, implying that the disorders of coppersmiths could also extend to other craftsmen working with metal, including blacksmiths. Indeed, Ramazzini is keen to note in that chapter he considers ‘those who work with copper in their city workshops, not copper miners who I have already mentioned in the first chapter on metal miners’.^8^

Finally, it should be noted that in his masterpiece, Ramazzini describes another occupational disease relevant to otolaryngologists. Voice disorders among occupational voice users, that is, workers whose voice is essential to their job and who rely on their voice as a means of employability (e.g. teachers, singers, actors), is an emerging problem, even though insurance companies recognise voice disorders less than occupational hearing disorders.^14^ In the chapter XXXVIII of *De Morbis Artificum*, the Italian physician describes ‘diseases that generally affect music teachers, singers and the like’^15^. Although including this category of workers was innovative and pioneering for his time, Ramazzini did not recognise that these problems were related to laryngeal alteration. Instead, he stated that ‘head colds and hoarseness are common ills in singers and actors, once again owing to the muscle tension that causes more lymph than necessary to be secreted by the salivary glands’.^8^ However, a modern reader should remember that, at that time, the role of the vocal folds and the larynx in general in phonation was not yet known, although contemporaneous contributions of the French naturalist Denis Dodart (1634–1707) were beginning to provide insight. Despite these limitations, the words of Bernardino Ramazzini appear pioneering for his time and represent an important milestone in the history of otology and laryngology.
